# Distribution of the Warmblood Fragile Foal Syndrome Type 1 Mutation (PLOD1 c.2032G>A) in Different Horse Breeds from Europe and the United States

**DOI:** 10.3390/genes11121518

**Published:** 2020-12-18

**Authors:** Simone Reiter, Barbara Wallner, Gottfried Brem, Elisabeth Haring, Ludwig Hoelzle, Monika Stefaniuk-Szmukier, Bogusława Długosz, Katarzyna Piórkowska, Katarzyna Ropka-Molik, Julia Malvick, Maria Cecilia T. Penedo, Rebecca R. Bellone

**Affiliations:** 1 Institute of Animal Breeding and Genetics, University of Veterinary Medicine Vienna, 1210 Vienna, Austria; simone.reiter1@gmail.com (S.R.); barbara.wallner@vetmeduni.ac.at (B.W.); gottfried.brem@bremkg.de (G.B.); 2 Central Research Laboratories, Museum of Natural History, 1010 Vienna, Austria; elisabeth.haring@nhm-wien.ac.at; 3 Department of Evolutionary Biology, University of Vienna, 1090 Vienna, Austria; 4 Institute of Animal Science, University of Hohenheim, 70599 Stuttgart, Germany; Ludwig.Hoelzle@uni-hohenheim.de; 5 Department of Animal Reproduction, Anatomy and Genomics, University of Agriculture in Kraków, al. Mickiewicza 24/28, 30-059 Kraków, Poland; monika.stefaniuk-szmukier@urk.edu.pl (M.S.-S.); boguslawa.dlugosz@urk.edu.pl (B.D.); 6 Department of Animal Molecular Biology, National Research Institute of Animal Production, Krakowska 1, 32-083 Balice, Poland; katarzyna.piorkowska@izoo.krakow.pl (K.P.); katarzyna.ropka@izoo.krakow.pl (K.R.-M.); 7 Veterinary Genetics Laboratory, School of Veterinary Medicine, University of California, Davis, CA 95616, USA; jzcollins@ucdavis.edu (J.M.); mctorrespenedo@ucdavis.edu (M.C.T.P.); 8 Population Health and Reproduction, School of Veterinary Medicine, University of California, Davis, CA 95616, USA

**Keywords:** PLOD 1, warmblood fragile foal syndrome, Arabians, museum sample

## Abstract

Warmblood fragile foal syndrome (WFFS) is an autosomal recessive disorder caused by a single nucleotide variant in the *procollagen-lysine-2-oxoglutarate-5-dioxygenase 1* gene (PLOD1:c.2032G>A, p.Gly678Arg). Homozygosity for the PLOD1 variant causes an Ehler-Danlos-like syndrome, which has to date only been reported in warmblood breeds but the WFFS allele has been also detected in the Thoroughbred. To investigate the breed distribution of the WFFS allele, 4081 horses belonging to 38 different breeds were screened. In total, 4.9% of the horses representing 21 breeds carried the WFFS allele. The affected breeds were mainly warmbloods, with carrier frequency as high as 17% in the Hanoverian and Danish Warmblood. The WFFS allele was not detected in most non-warmblood breeds. Exceptions include WFFS carriers in the Thoroughbred (17/716), Haflinger (2/48), American Sport Pony (1/12), and Knabstrupper (3/46). The origin of the WFFS allele remains unknown. The Arabian breed and specifically the stallion Bairactar Or. Ar. (1813), whose offspring were reported to have a similar phenotype in the 19th century, were hypothesized as the origin. DNA from a museum sample of Bairactar Or. Ar. showed that he did not carry the mutated allele. This result, together with the genotypes of 302 Arabians, all homozygous for the reference allele, does not support an Arabian origin of the WFFS allele. Our extensive survey shows the WFFS allele to be of moderate frequency and concern in warmbloods and also in breeds where it may not be expected.

## 1. Introduction

Ehler-Danlos syndrome (EDS) is a heterogeneous group of connective tissue disorders in humans caused by mutations in at least 20 different genes [1]. Characteristic symptoms of EDS are fragility of the soft connective tissues with widespread manifestations in skin, ligaments and joints, blood vessels, and internal organs [2]. In the last several decades, EDS-like syndrome was described in a broad range of mammals [3] as well as in several horse breeds including heavy horses [4], warmblood horses [5,6,7,8], Arabians [9,10], Quarter horses [11,12,13,14,15,16,17,18,19,20,21,22,23] and Thoroughbreds [24].

Two single nucleotide variants (SNVs) are documented as causal for EDS-like syndromes in horses. The first, hereditary equine regional dermal asthenia (HERDA), is a degenerative skin disease occurring in the Quarter horse and Quarter Horse-related breeds caused by a missense mutation (PPIB: c.115 G>A) in the cyclophilin B (PPIB) gene [25].

The second EDS-like syndrome was named Warmblood fragile foal syndrome type 1 (WFFS) because it was initially identified in warmblood breeds. It is proposed to be an autosomal recessive disorder caused by a SNV in the procollagen-lysine-2-oxoglutarate-5-dioxygenase 1 gene (PLOD1:c.2032G>A, p.Gly678Arg) [26,27]. Homozygosity for this PLOD1 variant (WFFS allele) is thought to be incompatible with extra-uterine life with a total of 18 cases described to date in the literature [26,27,28,29]. The predominant manifestation is death during the later stages of gestation (*n* = 4) or live births with foals being non-viable or euthanized because of poor prognosis (*n* = 14) [28]. Affected foals show skin abnormalities, including hyperextensible and abnormally thin skin that result in open lesions, as well as abnormal flexibility of digital joints at the time of birth. However, the role of the WFFS allele in embryonic loss is yet unknown.

Screening of multiple horse breeds during the commercialization of this test at the Veterinary Genetics Laboratory, University of California, Davis, USA (VGL) identified the presence of the WFFS allele in a broad range of warmblood breeds from different countries and also in the Thoroughbred. The presence of WFFS carriers was further investigated in the Thoroughbred and was determined to be at low frequency (2.4% in the 716 horses evaluated). Further, the WFFS allele was not associated with catastrophic breakdown [30]. Most recently, the WFFS allele frequency was estimated to be 14% in Hanoverians [29]. Pedigree analyses of WFFS carriers in warmbloods suggested that the variant could trace to the English Thoroughbred stallion Dark Ronald, a formative ancestor in many German warmblood lines [31]. However, DNA isolated from the preserved skin of Dark Ronald showed that he was homozygous for the wild-type PLOD1 variant, thus rejecting this hypothesis [32]. To date, the exact origin of WFFS is unclear. The famous imported Arabian stallion Bairactar Or. Ar. (1813) has also been hypothesized to be the founder of the WFFS allele, as offspring that resulted from inbreeding to him were reported to have a disease, similar to WFFS [9].

The stallion Bairactar Or. Ar. was imported from the Middle East in 1817, to the German stud in Weil. Until the first half of the 20th century the sire line was maintained mainly at Weil, but recently Bairactar Or. Ar.’s descendants are found in many warmblood breeds and bloodlines. Among these is the stallion Amurath Sahib who was the most influential Arabian sire line used in Poland. To investigate whether Bairactar Or. Ar. was a potential founder of WFFS, we isolated DNA from his museum remains. We also isolated DNA from other Arabian horses to test for the presence of the WFFS allele among Arabians from diverse lines including those that trace to Amurath Sahib.

In this study, carrier frequency and distribution of the WFFS allele were examined in a diverse sample set of over four thousand individuals representing 38 different breeds, including several warmblood breeds. Warmbloods are a group of horses with medium size body types representing many breeds that primarily originated in Europe and that have known draft (“coldblood”), Thoroughbred, and Arabian (“hotblood”) ancestry. They are registered with various breed registries that are characterized by open studbook policy, studbook selection, and are often bred for competitive equestrian sports. In addition to the warmbloods, Thoroughbreds, coldbloods, and ponies were also investigated. Horses were selected to cover a broad range of breeds and samples from both Europe and the United States to assess allele frequencies and distribution of the WFFS allele among breeds. Identification and estimation of breed-specific allele frequencies can guide breed genetic testing recommendations related to WFFS.

## 2. Materials and Methods

### 2.1. Sample Description Population Screening

Hair and blood samples of 4081 horses from 38 different breeds were analyzed in this study. The collection includes Thoroughbreds, warmblood horse breeds, Quarter Horses, breeds derived from warmbloods, and breeds in which warmbloods are allowable crosses. The sample set was also supplemented by a large number of Arabians (302), a selection of baroque horses, such as Friesians and Lippizans, and draft and pony breeds (Table 1).

A total of 2127 DNA samples representing 32 different breeds, banked at the UC Davis Veterinary Genetics Laboratory (VGL), were available for investigation. Samples were filtered for relatedness within breeds, based on one degree of separation. A total of 1238 horses were previously sampled for other research projects and stored in the Department of Animal Reproduction, Anatomy and Genomics, under the University of Agriculture in Krakow requirements (Ethical Agreement no. 00665 and 1173/2015). The sampled Arabians include representatives of descendants of Bairactar Or. Ar. (16 mares and 3 stallions) used in the Polish breeding program. The experimental protocol for Arabians was approved by the Animal Care and Use Committee of the Institute of Pharmacology, Polish Academy of Sciences in Krakow (no. 1173/2015).

The 716 Thoroughbred horses included in this study were used in Bellone et al., 2019 [30] and were included here for completeness of the dataset.

### 2.2. DNA Isolation and Genotyping of WFFS

DNA isolation and genotyping were performed with two different methods. In method A, DNA isolation from hair follicles was done as previously described in Locke et al. 2002 [33]. DNA was amplified for the PLOD1 c.2032G>A variant using the commercially available assay at the UC Davis VGL (https://vgl.ucdavis.edu/test/wffs). To ensure accurate genotyping, assays were run with three positive controls (one for each genotype) and one negative control. Positive controls genotyped as expected and negative controls did not yield detectable PCR product.

In method B, DNA was isolated from whole blood or hair follicles using Sherlock AX (A&A Biotechnology) according to the manufacturer’s protocol. The PLOD1 c.2032G>A variant was genotyped using a PCR-RFLP method. The primers for the PLOD1 gene were designed based on ENSECAG00000022842 (fwd: 5′-CTCGTGGTAGTGCGTGAGTC-3′ and rev: 5′-AGGGCCCAGCTTCCTCTT-3′) reference using PrimerInput3 (0.4.0). The endonuclease FauI, which only cuts the unmutated G-Allele but not the mutated A-Allele, was selected using NebCutter V2.0. PCR was performed using AmpliTaq Gold™ 360 Master Mix (Thermo Fisher Scientific, Waltham, MA, USA) according to the protocol with an annealing temperature of 57 °C. PCR fragments were digested with FauI (New England Biolabs, Ipswich, MA, USA) according to the protocol and visualized on a 4% agarose gel (G-Allele 102 and 64 bp; A-Allele 166 bp). Amplicons from all heterozygous and some homozygous samples were confirmed by Sanger sequencing using BigDye™ Terminator v3.1 Cycle Sequencing Kit (Thermo Fisher Scientific) and 3500×L Genetic Analyzers (Applied Biosystems, Foster, CA, USA; Thermo Fisher Scientific).

Allele and carrier frequencies were calculated for each breed and over all horses using Excel (Microsoft Office). Additionally, 95% confidence intervals were calculated for those breeds with more than 30 samples and for the overall sample set using the modified Wald Method in Graph Pad (https://www.graphpad.com/quickcalcs/confInterval2/) [34].

### 2.3. Historic DNA—Bairactar Or. Ar.

**Figure 1:**
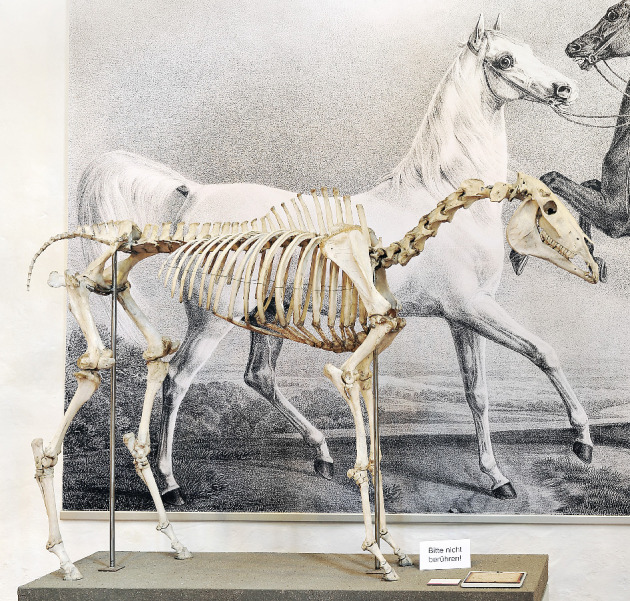
Skeleton of the Arabian stallion Bairactar Or. Ar. (1813–1838) in the Stud Museum Offenhausen in Germany (copyright: Stephan Kube). A tooth from this skeleton was used for DNA analysis.

A molar tooth of the stallion Bairactar Or. Ar. (1813–1838) was taken from his skeleton at the Stud Museum Offenhausen in Germany (Figure 1). To avoid contamination, DNA extraction was performed in the clean room of the Central Research Laboratories at the Natural History Museum in Vienna.

DNA was extracted from 1000 mg tooth powder using the GEN-IAL^®^ All-tissue DNA-Kit adjusting the manufacturer’s instructions (find details in File S1). Finally, DNA was dissolved in 30 μL nuclease-free water (Invitrogen™, Carlsbad, CA, USA). A final DNA concentration of 2.5 ng/µL was determined with Qubit™ dsDNA HS Assay Kit. No-template controls were included for the whole process of DNA isolation.

A 235 bp PCR product flanking the WFFS allele was amplified in 4 independent 50 µL reactions containing 1.75 µL DNA extract, 1 µM each of the primers PLOD1_B_fwd:5′-GTCACTCCACAAGGCACAAG-3′ and PLOD_1_B_rev: 5′-GTGGTAGTGCGTGAGTCGTC-3′, 0.25 mM of each dNTP, 2 mM MgCl2, 1× AmpliTaq Gold^®^ 360 Buffer, and 1.25 U AmpliTaq Gold^®^ 360 DNA Polymerase (Applied Biosystems™). PCR conditions were 5 min at 95 °C, followed by 40 cycles of 30 s at 95 °C, 30 s at 59 °C, 30 s at 72 °C, and a final extension of 7 min at 72 °C. PCR products were checked on 2% agarose gels, no product was visible.

Subsequently, 8 nested PCRs using 0.3 µM each of the primers PLOD1_C_fwd: 5′-AAACTGACGCTTCCTGTTGG-3′ and PLOD1_B_rev: 5′-GTGGTAGTGCGTGAGTCGTC-3′, resulting in a 143 bp product were performed in 25 µL reactions. Nested PCR reactions contained 3 µL PCR product of the first round as template, 0.25 mM of each dNTP, 1.5 mM MgCl2, 1× Buffer, and 0.5 U Taq Polymerase (Biozym, Hessisch Oldendorf, Germany). PCR conditions were 5 min at 95 °C, followed by 35 cycles of 30 s at 95 °C, 30 s at 59 °C, 30 s at 72 °C, and a final extension of 7 min at 72 °C. PCR products were visualized on 2% ethidium bromide-stained agarose gels. Negative controls, including the no-template controls from the first PCR rounds as input, were included.

As positive controls, two PCRs of known heterozygous horses using the primers PLOD1_B_fwd and PLOD_1_B_rev were done on a separate day to avoid contamination, following the nested PCR protocol described above.

DNA bands of the nested PCRs and the amplicons of the two controls were purified with Qiagen Gel-extraction-kit on different days and sent for Sanger sequencing at LGC genomics in Berlin (Germany).

All 20 sequencing traces (8 products, fwd and rev each from Bairactar Or. Ar.; 2 products, fwd and rev each from heterozygous controls) were analyzed using the software CodonCode Aligner 3.0.1.

## 3. Results

### 3.1. Distribution of the WFFS Allele

This study includes WFFS genotyping results of 4081 horses representing 38 different breeds, as summarized in Table 1. In total, 200 horses (4.9%) in our dataset carried the WFFS allele. The WFFS allele was detected in 21 breeds and carrier frequency ranged from 0% in most non-warmblood horse breeds (with the exception of Thoroughbred and Haflinger) to over 17% in the Hanoverian and Danish Warmblood. Among warmbloods, the WFFS allele was present in 17 of 19 breeds studied. It was not detected in the Swedish Warmblood (*n* = 16) and the Zangersheide Warmblood (*n* = 10), but the sample numbers of these breeds were small. The average carrier frequency across the 1610 warmblood horses tested in this study was 11%.

In addition to warmbloods, the WFFS allele was identified in the American Sport Pony, one of twelve horses examined was a carrier, and in the Knabstrupper with a carrier frequency of 6.5%. Two of 48 Haflingers were also identified as carriers (carrier frequency 4.2%). In reviewing pedigrees, a common ancestor among these two carriers was not identified in as far back as six generations.

The occurrence of WFFS carriers in Thoroughbreds has been shown previously to be 2.4% in Bellone et al., 2019 [30], but all 146 Thoroughbreds from Poland genotyped in this study were homozygous for the reference allele (PLOD1:c.2032G).

The WFFS allele was not detected in pony breeds such as the Shetland Pony, Hucul, Polish Konik, and the Norwegian Fjord. It was also absent in the tested draft breeds (Polish Heavy Draft and Shire), Quarter Horse, Appaloosa, and Baroque-type breeds like the Friesian, Friesian crosses, and Lipizzan. Akhal Teke, Arabian, and the tested gaited breeds (Tennessee Walker and Rocky Mountain Horse) also exclusively showed the reference allele.

**Table 1:** Warmblood fragile foal syndrome (WFFS) genotyping summary for all investigated breeds with the calculated carrier frequency, allele frequency, and 95% confidence interval for allele frequency. A short breed information is given for all breeds where carriers were detected.

Breed	Total	Carriers	Carrier Frequency %	Allele Frequency %	95% CI of WFFS Allele Frequency	Breed Information
Akhal Teke	35	0	0	0		
American Sport Pony	*12*	*1*	*8.33*	*4.17*		Warmblood-derived, studbook not closed
American Warmblood	57	8	14.04	7.02	3.41 to 13.43	Warmblood
Appaloosa	43	0	0	0		
Arabian	302	0	0	0		
Baden-Württemberger	*3*	*1*	*33.33*	*16.67*		Warmblood
Belgian Sport Horse	*10*	*1*	*10.00*	*5.00*		Warmblood
Belgian Warmblood	44	5	11.36	5.68	2.14 to 12.93	Warmblood
Canadian Warmblood	*29*	*3*	*10.34*	*5.17*		Warmblood
Danish Warmblood	127	22	17.32	8.66	5.74 to 12.82	Warmblood
Dutch Warmblood	249	19	7.63	3.82	2.42 to 5.92	Warmblood
Friesian	197	0	0	0		
Friesian Cross	72	0	0	0		
Haflinger	48	2	4.17	2.08	0.12 to 7.74	Origin in Europe with influence from several breeds including Arabians, studbook not closed until 1946
Hanoverian	283	49	17.31	9.01	6.90 to 11.67	Warmblood
Polish Heavy Draft	209	0	0	0		
Hessen	*2*	*1*	*50.00*	*25.00*		Warmblood
Holsteiner	132	11	8.33	4.17	2.26 to 7.39	Warmblood
Hucul	146	0	0	0		
Knabstrupper	46	3	6.52	3.26	0.72 to 9.55	Baroque type; Warmblood crosses allowed, studbook not closed
Lesser Poland Warmblood	157	3	1.91	0.96	0.19 to 2.91	Warmblood
Lippizan	42	0	0	0		
Norwegian Fjord	42	0	0	0		
Oldenburg	219	34	15.53	7.76	5.58 to 10.68	Warmblood
Polish Konik	96	0	0	0		
Quarter Horse	112	0	0	0		
Rheinland	12	2	16.67	8.33		Warmblood
Rocky Mountain Horse	89	0	0	0		
Selle Français	52	3	5.77	2.88	0.62 to 8.50	Warmblood
Shetland Pony	40	0	0	0		
Shire	39	0	0	0		
Silesian Horse	96	12	12.50	6.25	3.51 to 10.71	Warmblood
Swedish Warmblood	*16*	*0*	*0*	*0*		
Tennessee Walker	39	0	0	0		
Thoroughbred *	146	0	0	0		
Thoroughbred **	716	17	2.37	1.19	0.73 to 1.91	Arabian, Barb, and Turkoman ancestry
Trakehner	64	1	1.56	0.78	0.01 to 4.73	Warmblood
Westfalen	47	2	4.26	2.13	0.12 to 7.89	Warmblood
Zangersheide	*11*	*0*	*0*	*0*		
**total**	**4081**	**200**	**4.90**	**2.47**	**2.16 to 2.84**	

* Thoroughbreds sampled in Poland and firstly published in this article. ** Thoroughbreds already published in Bellone et al., 2019 [30]. Data in italic font denote breeds with small sample sizes (*n* < 30). Values for the total data set are given in bold.

### 3.2. Historic DNA–Bairactar Or. Ar.

**Figure 2:**
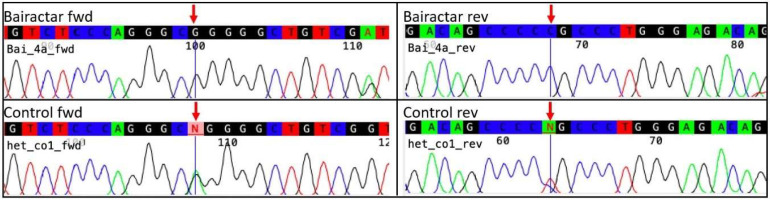
Sanger sequencing traces of Bairactar Or. Ar. (**upper panels**) and one heterozygous control horse (**lower panels**). The electropherograms correspond to sequences obtained with forward (**left side**) and reverse (**right side**) primers. The position of the WFFS single nucleotide variant (SNV) (procollagen-lysine-2-oxoglutarate-5-dioxygenase 1 gene (PLOD1) c.2032G>A) is marked with a red arrow.

The WFFS allele was not detected in any of the 16 sequences (8 fwd and 8 rev) generated out of DNA extracted from a tooth of Bairactar Or. Ar. (Figure 2). For two control horses, we confirmed that they were heterozygous carriers. Based on these results, we assume that the DNA extracted from the tooth is homozygous for the G-Allele (reference allele) at position PLOD1 c.2032. All 20 sequences are shown in File S1.

## 4. Discussion

The objective of this study was to determine the presence and frequency of the WFFS allele in diverse horse breeds. Based on our large dataset, which consisted of 4081 horses from 38 different breeds, we confirmed that, with exception of the Thoroughbred and the Haflinger, WFFS mainly occurs in warmblood breeds or those deriving from or allowing outcrosses to warmbloods.

It has been reported that the variant within PLOD1 that converts the amino acid in position 678 in the protein chain from Gly to Arg was carried by 11% of a small warmblood test population. Further pedigree analyses showed that WFFS might segregate among Hanoverian, Selle Français, Dutch Warmblood, Oldenburg, and Westphalian breeds [27]. The same carrier frequency of 11% was initially determined for 374 warmblood samples from Brazil in Dias et al., 2019 [35] and further confirmed in the present study for 1610 warmbloods from Europe and the United States. We detected the WFFS allele in 17 out of 19 tested warmblood breeds, with the highest estimated carrier frequency of 17.3% in the Hanoverian (283 horses) and the Danish Warmblood (127 horses). In a study of 849 Hanoverians, Metzger et al. 2020 [29] reported an allele frequency of 14%, making the expected carrier frequency 24%, which is notably higher than any of the other breeds investigated here. Pedigree analyses of Hanoverian WFFS carriers identified one stallion of the traditional sire line F/W as a most recent common ancestor. This stallion, born in 1861, maybe one of the major contributors to the spread of the WFFS allele in warmblood populations [29]. In our study, similarly high numbers of carriers as in the Hanoverian were detected in the Oldenburger (15.5%, 219 horses tested) and the American Warmblood (14.0%, 57 horses tested). The Silesian horse, a heavy Polish Warmblood breed developed from old Oldenburger bloodlines and recently influenced by the Thoroughbred, showed a slightly lower carrier frequency (12.5%, 96 horses tested). It should be noted that the sample size of the two warmblood breeds with no carriers detected was low (Swedish Warmblood *n* = 16, Zangersheide Warmblood *n* = 10) and, therefore, additional horses from these breeds should be evaluated before any conclusions on the presence of the WFFS allele can be drawn.

In addition to the traditional warmblood breeds, the WFFS allele was also detected in one American Sport Pony and three Knabstruppers. Given the warmblood influence in these breeds, this finding was not surprising. Specifically, crossing to warmblood horses is allowable for Knabstrupper. The American Sport Pony was recently derived in the United States from several breeds including warmblood horses. We also identified two Haflingers with a single copy of the WFFS allele, which was unexpected. Inspection of the pedigree records did not identify a common ancestor of these two individuals. However, one of the carriers had six unknown ancestors listed in the six-generation pedigree. The Haflinger breed was developed in Austria and Northern Italy with the foundation sire foaled in 1874, an offspring of a half Arabian stallion. The stud book was closed in 1946, whereby it is possible that prior to this, an infusion of European warmblood or Thoroughbred bloodlines could account for the WFFS allele in the Haflinger breed. Yet, so far the source of the WFFS allele in the Haflinger remains unknown, and further evaluation of the genetic contribution by other breeds to the Haflinger remains to be investigated.

Thoroughbred was the only non-warmblood breed in which the WFFS allele was previously described. In Bellone et al. 2019 [30] a carrier frequency of 2.4% in 716 Thoroughbreds was reported and it was shown that there was no association between the WFFS allele and catastrophic breakdown in this breed. In contrast to this result, we could not identify any carriers among the 146 Thoroughbreds from Poland genotyped for this study. This is likely best explained by the relatively low frequency overall in the Thoroughbred and the smaller sample size utilized for the Polish samples here compared to the previous work. Additionally, regionally restricted selection of samples may also explain why no carriers were detected in the Polish Thoroughbred sample set, as these may represent distinct subpopulations. Nevertheless, there is evidence for the origin of WFFS in the Thoroughbred because of the occurrence of the WFFS allele in this breed and the large influence of Thoroughbreds in warmblood breeds where the allele mainly occurs. However, the suggestion of Wobbe et al., 2019 [31] that Bay Ronald XX or his son Dark Ronald XX represent the common ancestor of all carriers could not be confirmed in Metzger et al., 2019 [29]. Indeed, Dark Ronald XX has been excluded as the founder in Zhang et al., 2020 [32] through DNA testing of skin remains.

We investigated the possibility that the Arabian breed was the origin of WFFS, which is based on writing from 1855 where symptoms similar to WFFS were described in foals inbred to Bairactar Or. Ar. [9]. Given that both the Thoroughbred and the Haflinger, two breeds with identified WFFS carriers, were influenced by Arabian horses, we sought to investigate this breed as the origin of the WFFS allele. Through DNA testing of historic remains, we found no evidence that the influential Arabian stallion Bairactar Or. Ar. (1813) was a carrier of the WFFS allele. Therefore, the hypothesis that Bairactar Or. Ar. spread the WFFS allele in Europe can be rejected. Nevertheless, it is still possible that a de novo germline mutation happened in Bairactar Or. Ar.’s son Amurath (*1829), from whom no remains were available for genotyping in this study. Further support of a non-Arabian origin of WFFS are the genotyping results of this study, wherein no carriers were identified among 302 Arabians tested. Thus, the likelihood of an Arabian origin of the WFFS allele is low, although given the presence of the WFFS allele in Haflinger, thought to have descended from a half Arabian stallion, this hypothesis cannot be definitively ruled out at this time.

The distribution of the WFFS allele appears to be restricted to warmblood horse breeds, the Thoroughbred, and a few other breeds that are likely derived from these. We did not detect carriers in Quarter Horses, Appaloosa, and gaited horses like the Tennessee Walker, which are breeds with a known Thoroughbred influence, suggesting these breeds are likely clear of WFFS caused by the PLOD 1 variant. In a broad range of pony and coldblooded breeds included in this study, the WFFS allele was not present in any of them, which might reflect their different origins. For example, Huculs and Polish Koniks represent primitive breeds described as feral. While Koniks are considered as Tarpan relics [36], Huculs originate from Carpathian Mountains horses [37]. The Polish Heavy Drafts arose after the Second World War and are based on local mares of coldblooded type crossed with imported breeding stock, mainly Ardennais and Bretons [38]. Given the results of this study, we propose that it is unlikely that Shetland Ponies, Polish Koniks, Norwegian Fjords and Huculs, Polish Heavy Drafts, and Shire horses will have WFFS caused by the PLOD1 variant. Nevertheless, in some instances, a limited number of samples were available. Six breeds had fewer than 30 samples available for testing (Table 1) and thus in these cases, further investigation is warranted to get a more accurate estimate of allele frequency [39], especially in instances where WFFS is suspected in an affected foal.

Our data further support the use of genetic testing for WFFS in warmblood breeds to prevent the production of affected foals. DNA test results can be utilized to avoid mating between carriers while still retaining carriers in the breeding program to maximize diversity and the production of other desirable traits. Given the presence of the WFFS allele in the Knabstrupper and American Sport Pony, it is also advisable to test breeding animals from warmblood-derived breeds or from breeds where crossing to warmbloods is allowed. DNA screening should even be performed in those breeds that may have genetic influence from warmblood breeds, as exemplified by the occurrence of the WFFS allele in the Haflinger breed. Screening for genetic diseases such as WFFS is not only of significant economic importance but it is also essential to avoid animal suffering and thus proper use of genetic testing in marker-assisted selection to prevent disease traits in horses cannot be over- emphasized.

## 5. Conclusions

Our comprehensive dataset reflects that WFFS is a concern in warmbloods and even occurs in breeds where it was not expected, like the Haflinger. While the origin of WFFS remains unclear, it is recommended to perform genetic tests even in non-warmblood related breeds, at least until there is more clarity about the occurrence of the WFFS allele.

## Supplementary Materials

The following are available online at https://www.mdpi.com/2073-4425/11/12/1518/s1, File S1: Detailed description of historic DNA analysis from Bairactar Or. Ar.
