# Supplementary material for: Distribution of the Warmblood Fragile Foal Syndrome Type 1 Mutation (PLOD1 c.2032G>A) in Different Horse Breeds from Europe and the United States
Source: Genes (Basel). 2020 Dec 18;11(12):1518. doi: 10.3390/genes11121518 (PMC7766603; doi:10.3390/genes11121518)
Supplement: Supplementary file 1 [file genes-11-01518-s001.pdf]

Supplementary File S1

Historic DNA Analysis of Bairactar Or. Ar.

Sample preparation, DNA Isolation, PCR and sequencing

A molar tooth of the stallion Bairactar Or. Ar. (1813–1838) was taken from his skeleton in the Stud Museum Offenhausen in Germany. DNA extraction was performed in the cleanroom of the laboratory at the Natural History Museum in Vienna, following standard procedures to avoid contamination. The surface of the tooth was removed by abrasion, to minimize environmental contamination. About 1000 mg of tooth got ground into powder with a Retsch MM400 grinding mill (30 Hz/30 sec), using a 10 ml zirconium oxide grinding jar and a 12 mm zirconium oxide grinding ball. The powder was transferred to a 5 ml DNA lo bind tube. For Decalcification of the tooth powder, 4.5 ml Decalcifier soft (containing 25% EDTA) was added. Tubes were incubated with rotation overnight at 4 °C. After 10 min centrifugation at 4000 rpm the supernatant was discarded and replaced by 4.5 ml fresh decalcifying solution. The tooth powder was dissolved by vortexing and again incubated with rotation overnight at 4 °C. This procedure was repeated two times. After decalcification, the powder was washed to remove all remains of EDTA by adding 4.5 ml nuclease free water to each sample and after vortexing, the tubes were centrifuged 10 min at 4000 rpm. The supernatant was discarded and the washing step was repeated two times.

DNA was extracted using the GEN-IAL® All-tissue DNA-Kit adjusting the manufacturer's instructions for DNA extraction from bones and teeth. 2.5 ml lysis buffer1, 250 µl lysis buffer2, 100 µl proteinase K and 75 µl 1 M dithiothreitol were added to the sample. The tube was incubated with rotation at 56 °C for 4 days. After 15 min centrifugation at 8000 rpm the clear supernatant was divided into two new 5 ml DNA lo bind tubes and 0.75 volume of lysis buffer 3 was added to each tube. After short vortexing the tube was incubated at −20 °C for 5 min, followed by centrifugation for 10 min at 13,000 rpm. Next, the supernatant was transferred to a new 5 ml DNA lo bind tube and the DNA was precipitated by adding 0.80 volume isopropanol and 15 min incubation at 4 °C. 1400 µl supernatant were transferred into a 1.5 ml DNA lo bind tube followed by centrifugation at 13,000 rpm. The supernatant was discarded and again 1400 µl supernatant from the previous step were added to the tube. This procedure was repeated until the whole volume was centrifuged. The pellet was washed with 300 µl cold 70% ethanol and centrifuged for 5 min at 13,000 rpm. Next, the ethanol was discarded and the pellet dried at room temperature. Finally, the DNA was dissolved in 30 µl nuclease free water. For the whole DNA Isolation procedure empty controls were included. DNA concentration was checked with Qubit™ *dsDNA HS Assay Kit*.

A 235 bp long product was amplified using in 4 different 50 µl reactions containing 1.75 µl DNA extract, 1 µM each of the primers PLOD1_B_fwd (5′-GTCACTCCACAAGGCACAAG-3′) and PLOD_1_B_rev (5′- GTGGTAGTGCGTGAGTCGTC-3′), 0.25 mM of each dNTP, 2 mM $MgCl_2$, 1 x AmpliTaq Gold® 360 Buffer, and 1.25 U AmpliTaq Gold® 360 DNA Polymerase (Applied Biosystems™). PCR conditions were 5 min at 95 °C, followed by 40 cycles of 30 sec at 95 °C, 30 sec at 59 °C, 30 sec at 72 °C, and a final extension of 7 min at 72 °C. PCR products were checked on 2% agarose gels, no product was visible.

Subsequently, 8 nested PCRs using 0.3 µM each of the primers PLOD1_C_fwd (5′-AAACTGACGCTTCCTGTTGG-3′) and PLOD1_B_rev (5′-GTGGTAGTGCGTGAGTCGTC-3′), resulting in a 143 bp product, were performed in 25 µl reactions. Nested PCR reactions contained 3 µl PCR product of the first round as template, 0.25 mM of each dNTP, 1.5 mM $MgCl_2$, 1 x Buffer, and 0.5 U Taq Polymerase (Biozym). PCR conditions were 5 min at 95 °C, followed by 35 cycles of 30 sec at 95 °C, 30 sec at 59 °C, 30 sec at 72 °C, and a final extension of 7 min at 72 °C. PCR products were visualized on 2% agarose gels. Empty controls, including the empty controls from the first PCR rounds as input were included for each PCR reaction.

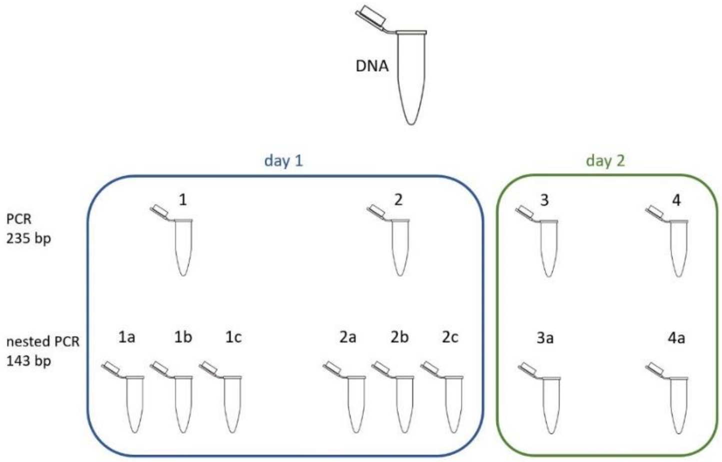

Figure S1. Setup PCR and nested PCR.

As a control two PCRs of known heterozygous horses using the primers PLOD1_B_fwd (5′-GTCACTCCACAAGGCACAAG-3′) and PLOD_1_B_rev (5′- GTGGTAGTGCGTGAGTCGTC-3′) were done following the protocol of the nested PCR above.

DNA bands of the nested PCRs and the amplicons of the two controls were purified with Qiagen Gel-extraction-kit on different days and sent for Sanger sequencing at LGC genomics in Berlin (Germany).

Sample preparation, DNA Isolation and the first PCR reaction were carried out in the cleanroom of the laboratory at the Natural History Museum in Vienna. The nested PCR was carried out in a molecular genetic lab at the Institute for Animal Breeding and Genetics at the University for Veterinary Medicine in Vienna.

All 20 sequences (8 products, fwd and rev each from Bairactar; 2 products, fwd and rev each from controls) were analysed with CodonCode Aligner 3.0.1.

Results

In all 16 sequences sequenced from the sample of ‘Bairactar’ the WFFS SNV PLOD1 c.2032G>A was not detected (Figure S2). In the two control horses we confirm that they are heterozygous carriers. Based on these results, we assume that the DNA extracted from the tooth is homozygous for the G-Allele at position PLOD1 c.2032.

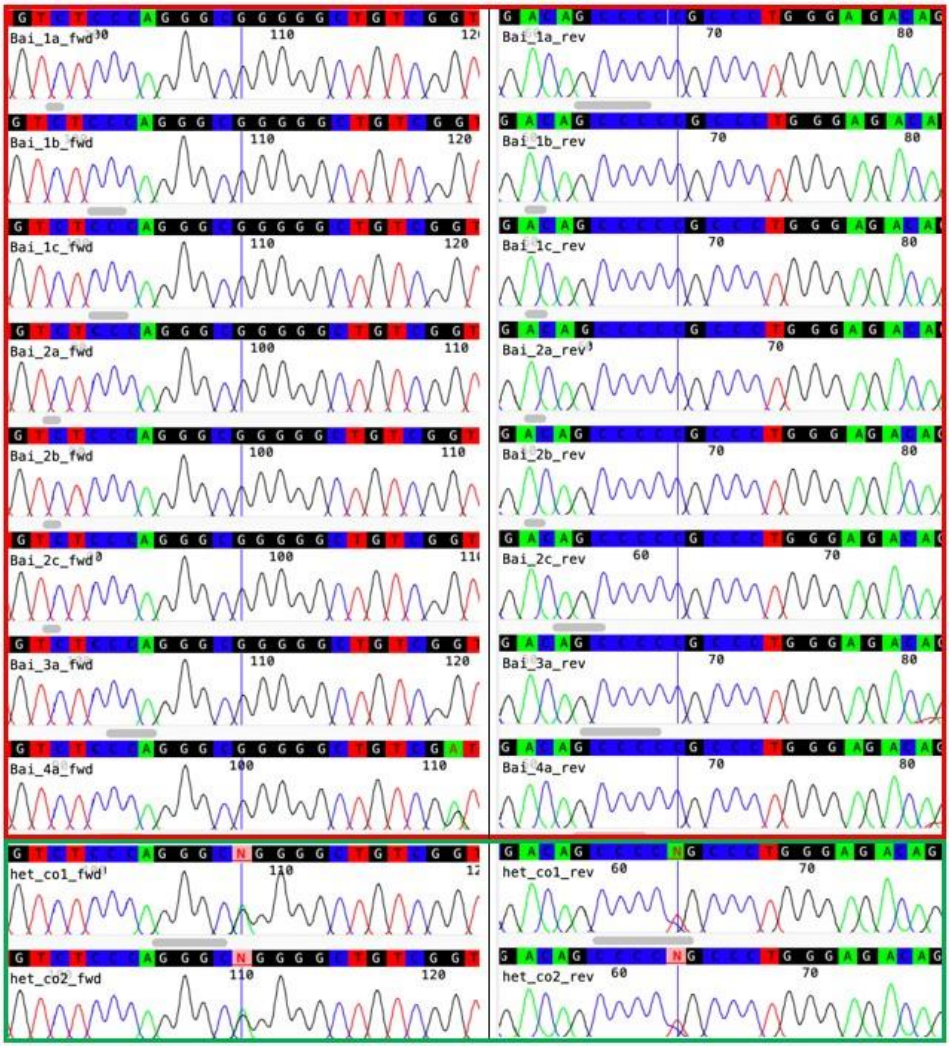

Figure S2. Sanger sequencing traces of Bairactar Or. Ar. (red box) and two heterozygous control horses (green box). The electropherograms correspond to sequences obtained with forward (left side) and reverse (right side) primers. The position of the WFFS single nucleotide variant (SNV) (procollagen-lysine-2-oxoglutarate-5-dioxygenase 1 gene (PLOD1) c.2032G>A) is marked with a blue line.
